# Supplementary figures and images for: The transcription factors Tfeb and Tfe3 are required for survival and embryonic development of pancreas and liver in zebrafish
Source: PLoS Genet. 2025 Jun 27;21(6):e1011754. doi: 10.1371/journal.pgen.1011754 (PMC12225984; doi:10.1371/journal.pgen.1011754)

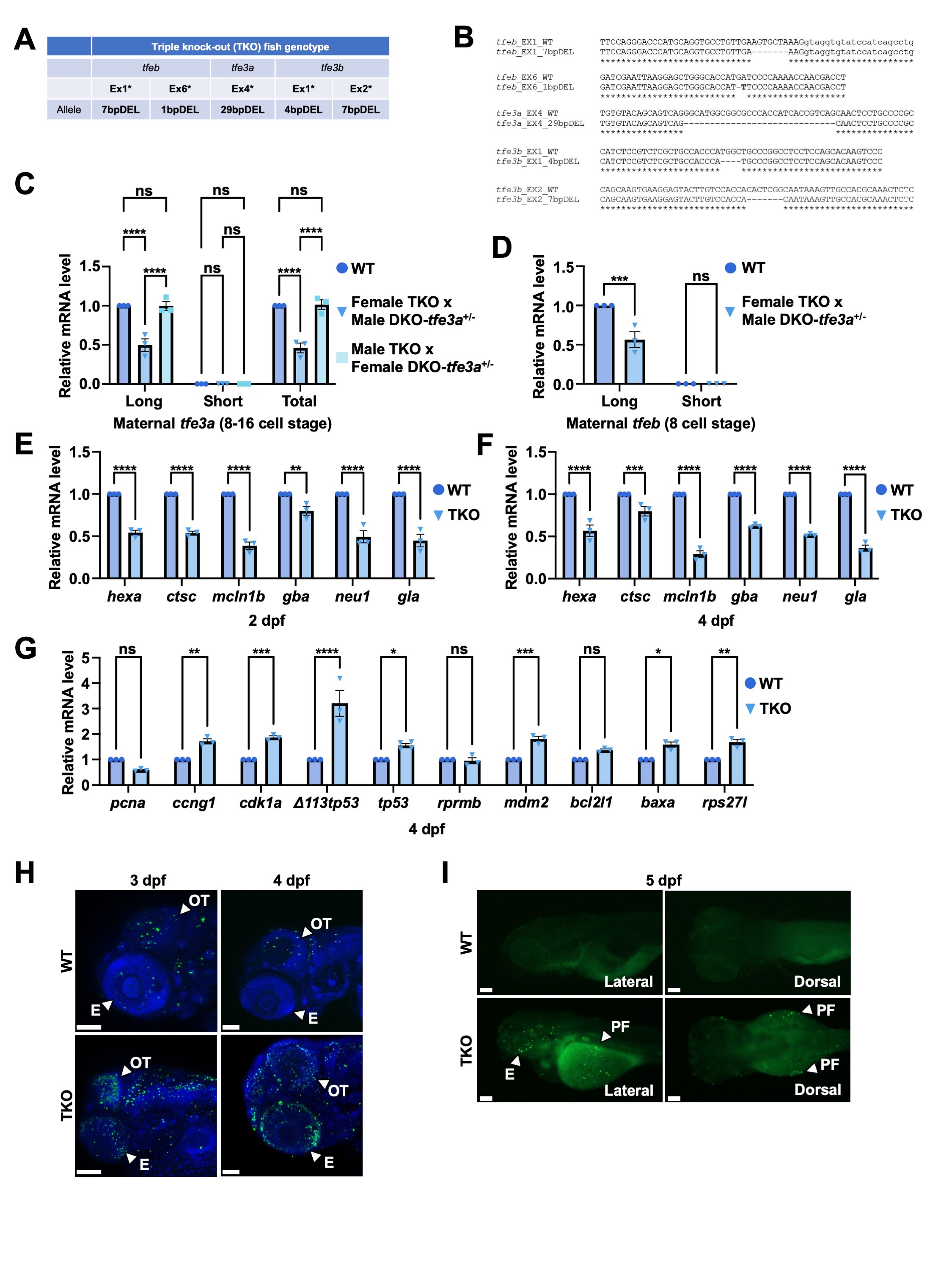

Supplement: S1 Fig — (A) Mutated alleles characterizing the genotype of TKO fish. (B) Genomic and mRNA regions around each gRNA target were amplified by PCR and then SANGER sequenced. Part of the sequenced regions surrounding each site has been compared to the WT sequences and is shown in each alignment. Lower case letters indicate intronic sequences, bold letters highlight single base insertions. Asterisks and dashes mark identical and deleted bases, respectively. (C) Real-time qPCR expression analysis of different tfe3a maternal forms at 8/16 cell stage in WT and embryos obtained from different crosses. The expression in WT embryos has been used as reference. (D) Relative expression analysis by real-time qPCR of long and short tfeb maternal forms at 8 cell stage in WT and embryos obtained from a cross of females TKO and males DKO-tfe3a+/-. (E-F) Analysis of lysosomal markers in TKO and WT embryos at 2 and 4 dpf. (G) Proliferation, cell cycle, pro- and anti-apoptotic expression markers at 4 dpf. Proliferation marker: pcna. Cell proliferation inhibiting factors: ccng1, p21, Delta113tp53. Pro-apoptotic genes: tp53, rps27l, mdm2 and baxa. Anti-apoptotic genes: bcl2l1. All the data represent means ± SEM, n = 3 independent experiments. Statistical significance was determined by using two-way ANOVA with Sidak’s multiple comparisons. ns, not significant, * < 0.05,** < 0.01, *** < 0.001, **** < 0.0001. (H-I) TUNEL assay in WT and TKO embryos at different developmental stages. (H) Confocal maximum projections of embryos showing TUNEL+ cells (green) and DAPI (blue) at 3 and 4 dpf. Lateral positions, anterior to the left. Scale bars, 100 μm. (I) Fluorescent stereomicroscope pictures of TUNEL staining (green) of 5 dpf embryos in lateral and dorsal orientation, anterior to the left. Scale bars, 150 μm. E, eye; OT, optic tectum; PF, pectoral fins. (TIFF) [file pgen.1011754.s001.tiff]

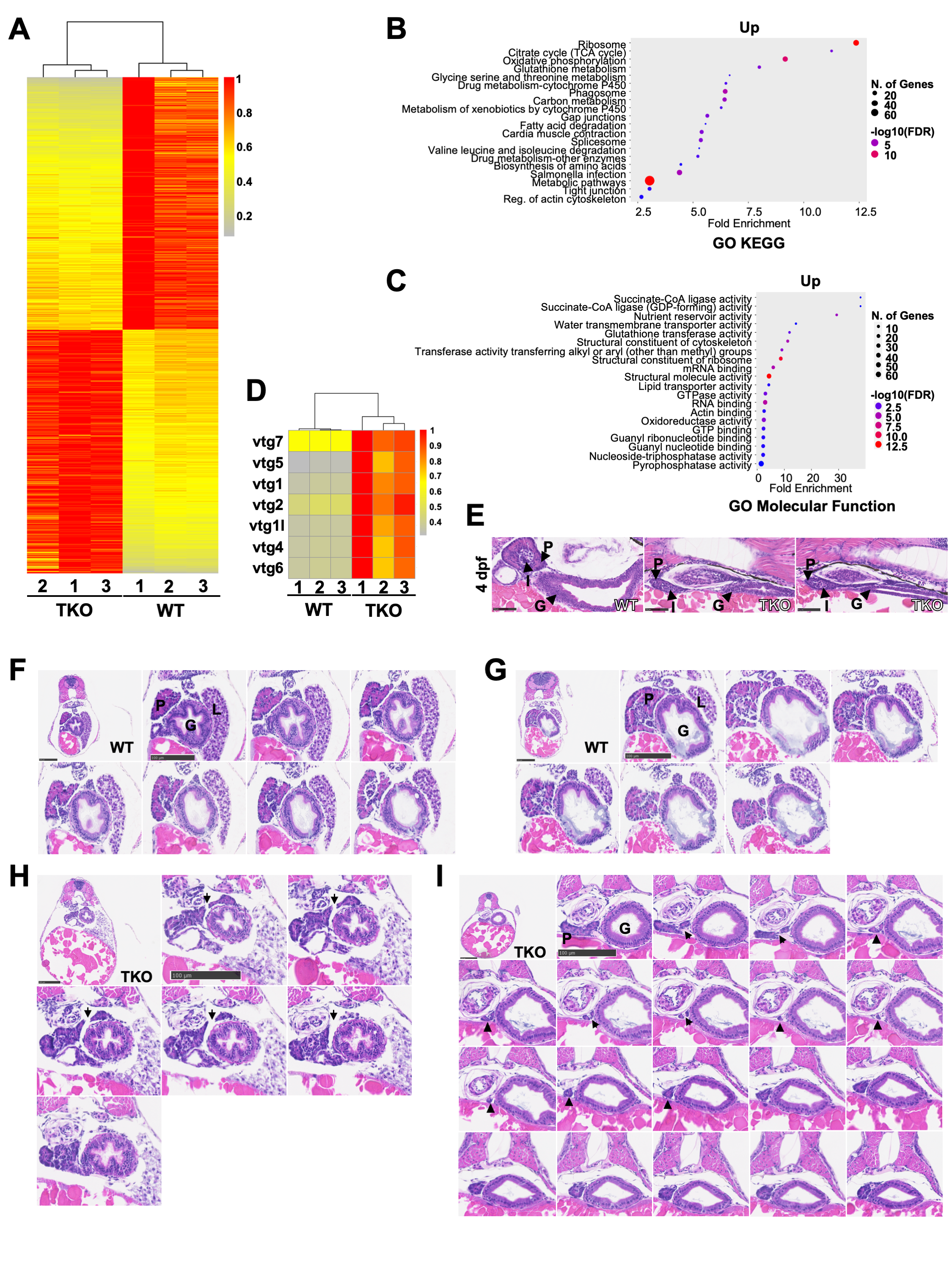

Supplement: S2 Fig — (A) Heat map showing the relative abundance of 856 differentially expressed ranked proteins (fold change >1.5, p < 0.05, n = 3). The color key indicates the relative abundance of each protein (0 to 1.0) across 6 samples. (B-C) ShinyGO KEGG (B) and Molecular Function (C) term enrichment of proteins significantly upregulated in TKO mutants compared to WT siblings. FDR, false discovery rate. (D) Heat map showing the relative abundance of vitellogenin proteins in WT and TKO samples. (E) H&E staining of longitudinal sections showing the pancreas and gut in the trunk of paraffin embedded WT and TKO embryos at 4 dpf. G, gut; I, islet; P, exocrine pancreas. Scale bars, 50 μm. (F-I) H&E staining of transversal sections showing the gastro-intestinal organs in the trunk of paraffin embedded WT (F-G) and TKO (H-I) 5 dpf embryos. Consecutive sections are shown in rostro-caudal order from left to right and from top to bottom of the sequence. Black arrows point to discontinuities in the exocrine pancreas tissue, black arrowheads indicate the central region of exocrine pancreas. P, exocrine pancreas; G, gut; L, liver. 5 μm sections. Scale bars, 100 μm. (TIFF) [file pgen.1011754.s002.tiff]

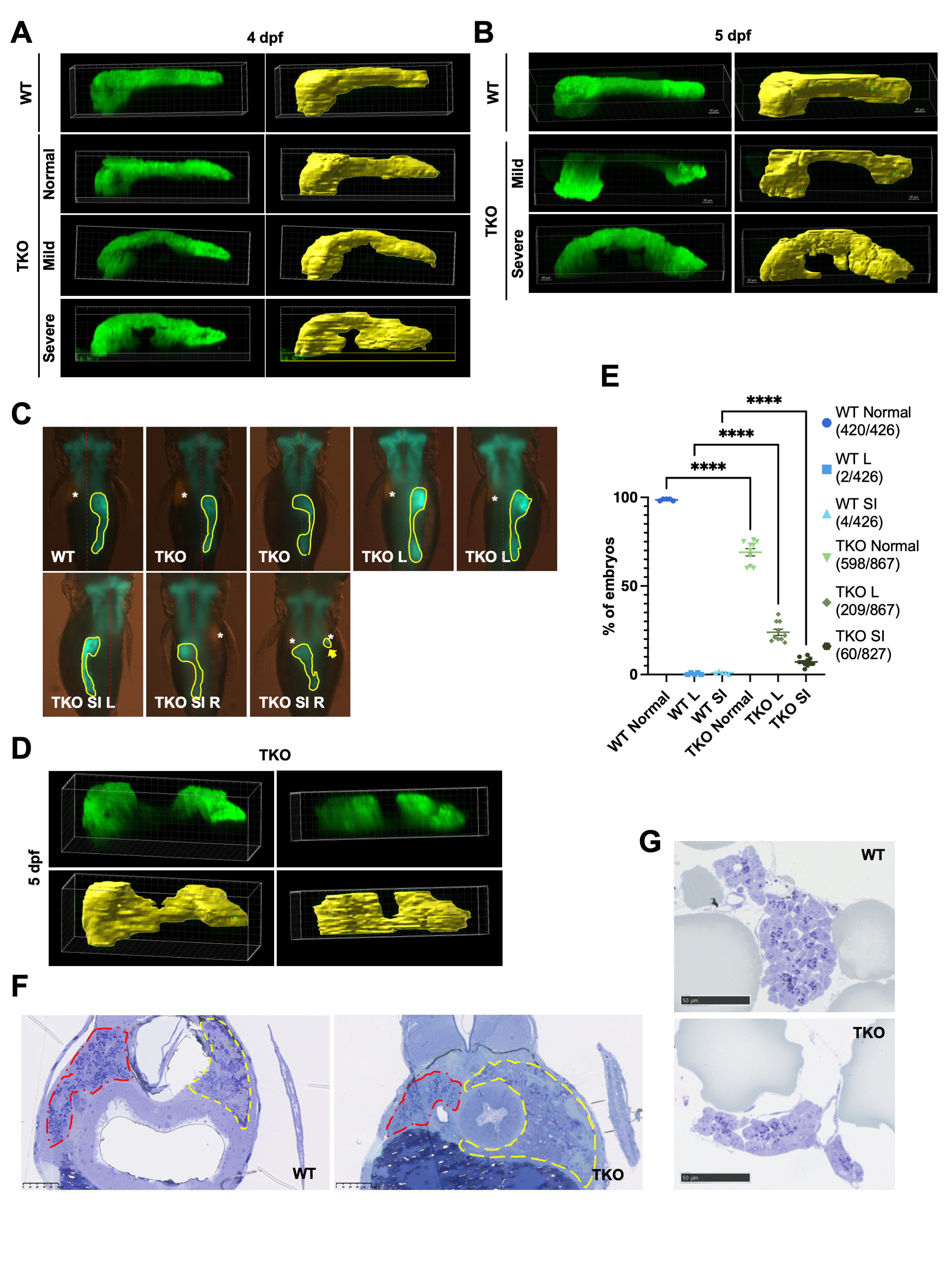

Supplement: S3 Fig — (A-B) Representative max-projections (green) and Imaris 3D reconstructions (yellow) of confocal Z-stacks of 2CLIP WT and TKO embryos at 4 and 5 dpf, respectively. Dorsal views, anterior to left. Scale bars, 30 μm. (C) Dorsal views of 4.5 dpf WT and TKO 2CLIP embryos. Red dashed lines indicate the midline of the embryos, pancreas have been highlighted with a yellow line. The yellow arrow points to GFP+ signal on the other side of the embryo near the liver (white asterisks), suggesting possible heterotaxia. (D) Representative max-projections (top) and corresponding Imaris 3D reconstructions (bottom) of confocal Z-stacks of TKO L embryos at 5 dpf. Dorsal views, anterior to the left. (E) Quantification of the percentage of embryos for each category in WT and TKO embryos at 4.5 dpf. L, lefty; R, righty; SI, Situs Inversus. (F-G) Toluidine staining of resin semithin transversal sections of 5 dpf embryos (F) and a portion of adult pancreas (G) showing the regions used for the TEM analysis. Liver and pancreas are highlighted in yellow and red, respectively. Scale bars, 50 μm. (TIFF) [file pgen.1011754.s003.tiff]

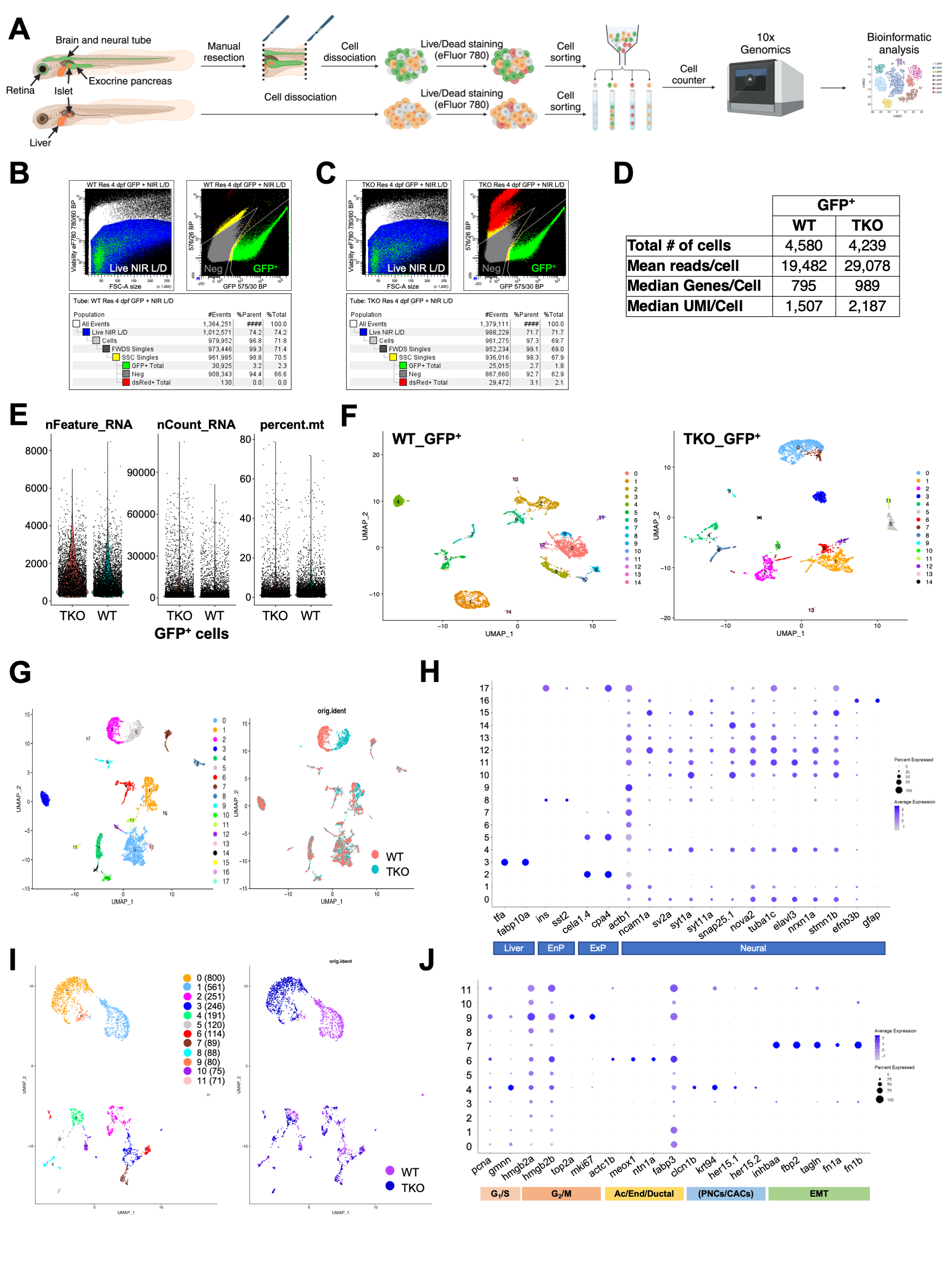

Supplement: S4 Fig — Single cell RNA-sequencing data from sorted GFP+ cells at 4 dpf. (A) Experimental workflow used for scRNA-seq experiments of 4 dpf embryos. (B-C) FACS isolation of the GFP+ cell population from 4 dpf WT and TKO 2CLIP embryos. (D) Table showing cell quality control metrics for the GFP+ cells generated by 10X Cell Ranger. (E) Violin plots showing quality control features (nFeature_RNA, nCount_RNA and percent.mito) for both, GFP+ WT and TKO data sets. (F) UMAPs showing clusterization of WT (left) and TKO (right) GFP+ cells. (G) UMAPs showing the projection of the clusters (left) and the two genotypes (right, WT and TKO) merged for the analysis. (H) Dot plot showing expression of liver, endocrine pancreas (EnP), exocrine pancreas (ExP) and neural markers in the different cluster population. (I) UMAPs showing clusters (left) and genotype (right) of the subset of the pancreas-related clusters. Numbers in parenthesis indicate the total number of cells for each cluster. (J) Dot plots showing expression of cell cycle (G1/S and G2/M), acinar/endocrine/ductal (Ac/End/Ductal), pancreatic Notch-responsive cells/centroacinar cells (PNCs/CACs), and epithelial-mesenchymal transition (EMT) markers in merged clusters shown in (I). In each dot plot, the size of the dot encodes the percentage of cells within a class, the color indicates the average level of expression (purple is high, grey is low). (TIFF) [file pgen.1011754.s004.tiff]

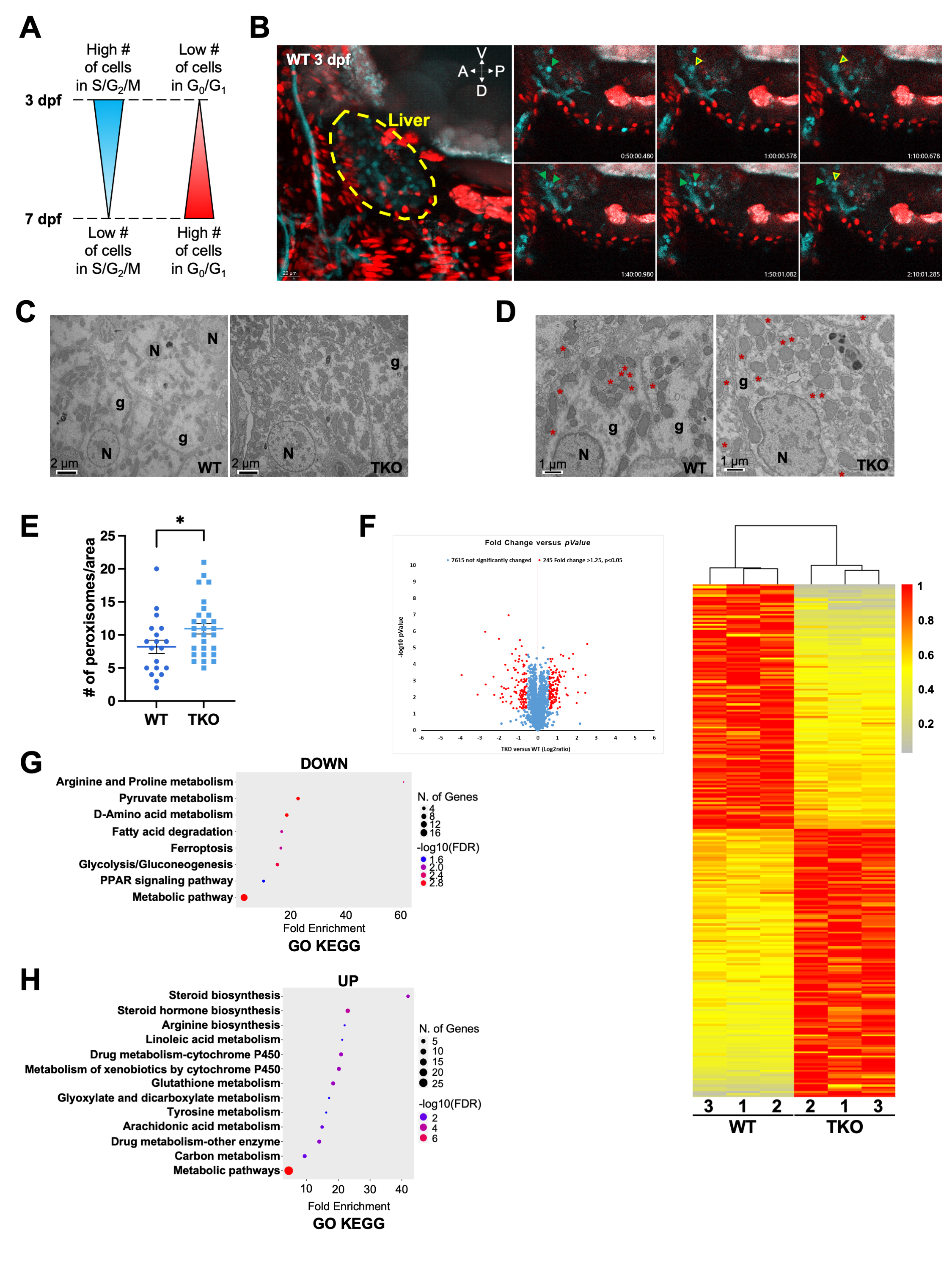

Supplement: S5 Fig — (A) Cartoon showing the proliferation rate during liver growth phase in WT embryos. Left lobe livers reach cellular homeostasis ~5–7 dpf. (B) (Left) Representative max-projection of confocal Z-stacks of a FUCCI WT embryo at 3 dpf. The dashed yellow line highlights the left lobe liver. Lateral views, anterior to the left and dorsal to the bottom. Scale bar, 20 μm. (Right) Pictures of the same single plane over time from each confocal Z-stack. Arrowheads point at the same cerulean+ cells over time; during cell division, arrowheads present an outline of a different color. Note that cerulean+ nuclei become brighter right before the beginning of mitosis. Numbers indicate the time of each frame, starting from 50 minutes. (C-D) Representative EM pictures of hepatocytes from WT and TKO adult fish. Red asterisks label cellular peroxisomes. N, nuclei; g, glycogen lacunae. Scale bar, 2 μm (C) and 1 μm (D). (E) Quantification of the number of peroxisomes/area (~150 µm2) in EM sections of WT and TKO adult livers. Data represents means ± SEM. Statistical significance was determined by unpaired two-tailed t-tests. * < 0.05. (F) (Left) A volcano plot showing 245 differentially expressed proteins between TKO and WT zebrafish (n = 3 each). X-axis represents log2 of fold changes, y-axis represents statistically significant p-value (-log10 of p-value, n = 3). Blue dots represent 7615 proteins fold change <1.5, red dots are 245 differentially expressed proteins with a fold change >1.5, p < 0.05. (Right) Heat map showing the relative abundance of the same 245 differentially ranked proteins. Color Key indicates the relative abundance of each protein (0 to 1.0) across the 6 samples. (G-H) ShinyGO KEGG term enrichment of those proteins significantly downregulated (G) and upregulated (H) in TKO mutants compared to WT siblings. FDR, false discovery rate. (TIFF) [file pgen.1011754.s005.tiff]

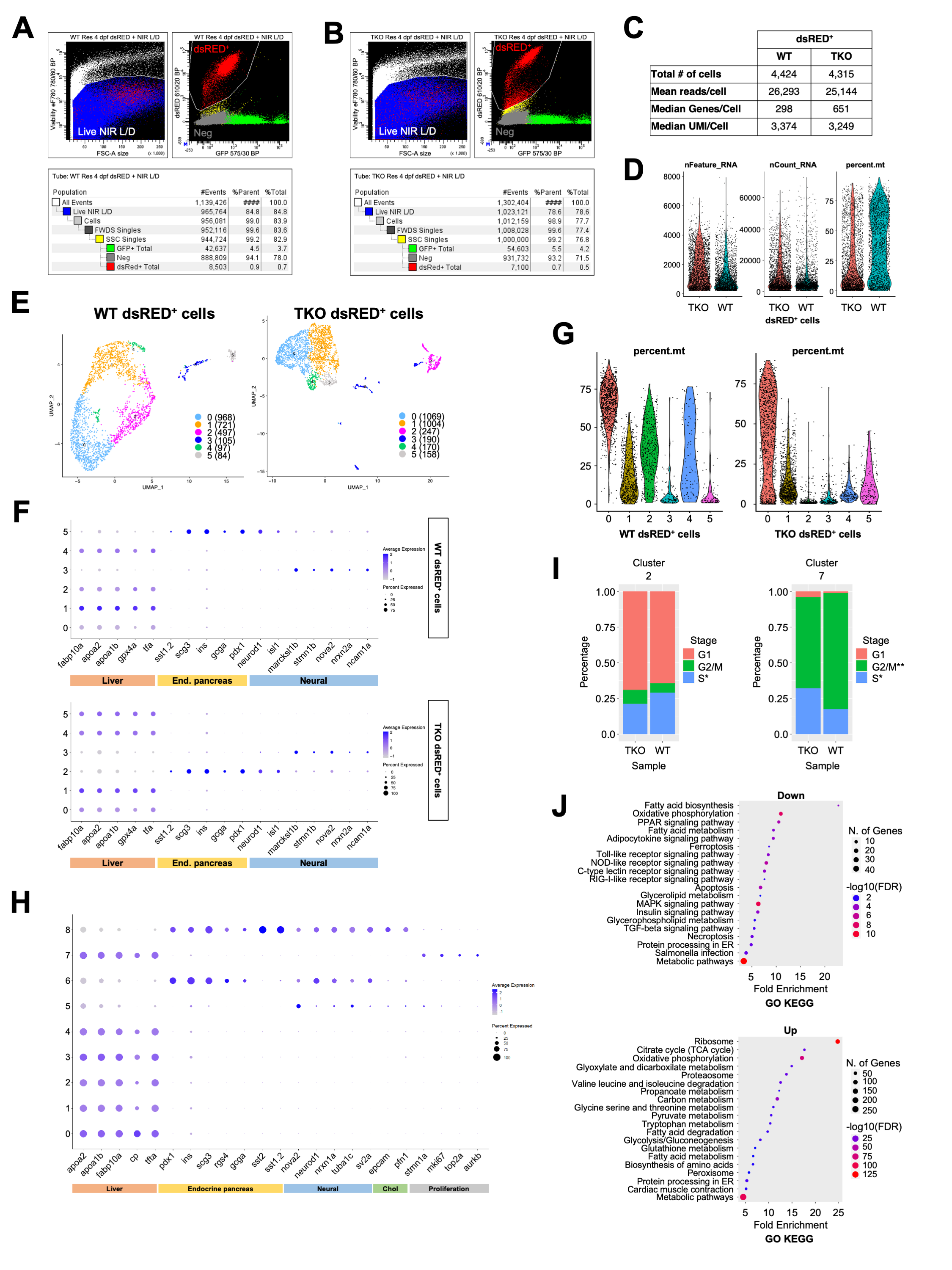

Supplement: S6 Fig — Single cell RNA-sequencing data from sorted dsRED+ cells at 4 dpf. (A-B) FACS isolation of the dsRED+ cell population from 4 dpf WT and TKO 2CLIP embryos. (C) Table showing cell quality control metrics for the dsRED+ cells generated by 10X Cell Ranger. (D) Violin plots showing quality control features (nFeature_RNA, nCount_RNA and percent.mt) for both, dsRED+ WT and TKO data sets. (E) UMAPs showing clusterization of WT (left panel) and TKO (right panel) dsRED+ cells. The total number of cells in each cluster in shown in parenthesis. (F) Dot plots showing expression of hepatic, endocrine pancreas, and neuronal markers in WT (top panel) and TKO (bottom panel) dsRED+ datasets. (G) Violin plots showing the quality percent.mt control feature of each cell cluster for both the dsRED+ WT (left) and TKO (right) data sets as shown in (E). (H) Dot plots showing expression of liver, endocrine pancreas, neural, embryonic cholangiocytes (Chol), and proliferation markers in merged clusters shown in Fig 6A. (I) Cell cycle analysis using Seurat CellCycleScoring function in WT and TKO hepatic cells. Statistical significance was determined using Fisher’s exact test. * < 0.05 and ** < 0.01. (J) Gene ontology enrichment analysis of downregulated (DOWN) and upregulated (UP) genes in TKO and WT liver clusters: clusters 1 vs 0 as shown in Fig 6C. In each dot plot, the size of the dot encodes the percentage of cells within a class, the color indicates the average level of expression (purple is high, grey is low). (TIFF) [file pgen.1011754.s006.tiff]

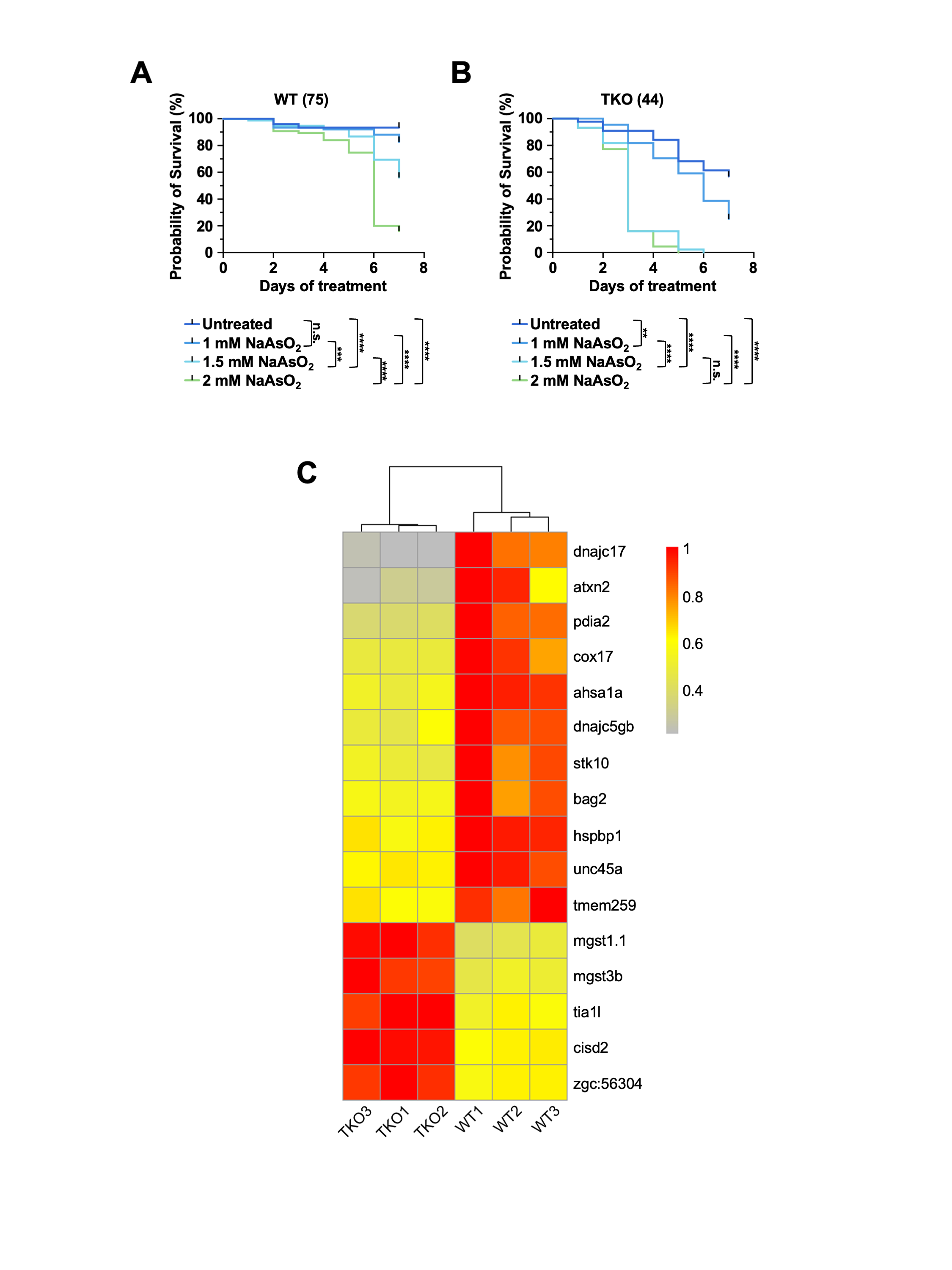

Supplement: S7 Fig — (A-B) Kaplan-Meier curves showing the survival rates of WT (A) and TKO (B) embryos following continuous exposure to different doses of NaAsO2 from 1 dpf stage. At each dose, untreated embryos were used as controls. The number of embryos per group is indicated in parenthesis. The same number of embryos has been used in each treatment. (Logrank tests: n.s., not significant; ** < 0.01; *** < 0.001, **** < 0.0001). Data show one representative experiment out of three independently performed. (C) Heat map showing the relative abundance of selected proteins at 5 dpf. The color key indicates the relative abundance of each protein (0.2 to 1.0) across the 6 samples. (TIFF) [file pgen.1011754.s007.tiff]
