# Supplementary material for: The transcription factors Tfeb and Tfe3 are required for survival and embryonic development of pancreas and liver in zebrafish
Source: PLoS Genet. 2025 Jun 27;21(6):e1011754. doi: 10.1371/journal.pgen.1011754 (PMC12225984; doi:10.1371/journal.pgen.1011754)
Supplement: S1 Table — Summary of all the different combinations of KO mutants analyzed and the survival observed during development and adulthood. In red are highlighted the combinations that presented embryonic lethality, in bold the ones that presented phenotypes during adulthood. (DOCX) [file pgen.1011754.s008.docx]

| **Table S1. Summary of mutants and their phenotypes** | | | | | | | | | | | |
| --- | --- | --- | --- | --- | --- | --- | --- | --- | --- | --- | --- |
|  |  | **tfeb** | | | **tfe3a** | | | **tfe3b** | |  |  |
| **Fish** | **Embryos** | **LONG maternal** | **LONG zygotic** | **SHORT zygotic** | **LONG maternal** | **LONG zygotic** | **SHORT zygotic** | **Maternal** | **Zygotic** | **Survival** | **Notes** |
| Female triple knock-out long x Male triple knock-out long (from DOI: 10.1126/sciadv.abp8321) | TKO long | NO | NO | **YES** | NO | NO | **YES** | NO | NO | **Triple knock-out adults alive and fertile. Delayed growth.** | tfeb and tfe3a short forms present low level of transcriptional activity which is probably enough to sustain the embryo development OR compensatory effect of other genes |
| Female TKO long+short x Male TKO long+short | TKO long+short | NO | NO | NO | NO | NO | NO | NO | NO | **Embryonically lethal** |  |
| **Female TKO long+short** x Male DKO-tfe3a+/- long+short | TKO | NO | NO | NO | NO | NO | NO | NO | NO | **Embryonically lethal** | tfe3a zygotic forms are NOT sufficient to induce embryo survival |
|  | DKO-tfe3a+/- | NO | NO | NO | NO | **YES** | **YES** | NO | NO | **Embryonically lethal** |  |
| **Female DKO-tfe3a+/- long+short** x Male TKO long+short | TKO | NO | NO | NO | **YES** | NO | NO | NO | NO | **Adult alive, but strongly reduced size if not separated** | Maternal tfe3a long forms alone are sufficient to induce embryo survival, but not a normal adult growth |
|  | DKO-tfe3a+/- | NO | NO | NO | YES | YES | YES | NO | NO | Adult alive and normal | tfe3a transcripts (maternal and zygotic) alone are  sufficient to induce embryo survival and normal adult growth |
| Female DKO-tfe3a+/- long+short X Male DKO-tfe3a+/- long+short | TKO | NO | NO | NO | **YES** | NO | NO | NO | NO | **Adult alive, but strongly reduced size if not separated** | Maternal tfe3a long forms alone are sufficient to induce embryo survival, but not a normal adult growth |
|  | DKO-tfe3a+/- | NO | NO | NO | YES | YES | YES | NO | NO | Adult alive and normal |  |
|  | DKO (tfeb and tfe3b nulls) | NO | NO | NO | YES | YES | YES | NO | NO | Adult alive and normal |  |
