# Supplementary material for: The transcription factors Tfeb and Tfe3 are required for survival and embryonic development of pancreas and liver in zebrafish
Source: PLoS Genet. 2025 Jun 27;21(6):e1011754. doi: 10.1371/journal.pgen.1011754 (PMC12225984; doi:10.1371/journal.pgen.1011754)
Supplement: S8 Table — (DOCX) [file pgen.1011754.s015.docx]

| **Table S8. List of oligos and gRNAs used in this study** | | | |
| --- | --- | --- | --- |
| Primer name | Application | Target gene | Sequence |
| tfeb_Ex1_gRNA | genome editing | tfeb Ex1 | taatacgactcactataggtgcctgttgaagtgctaagttttagagctagaa |
| tfeb_Ex6_gRNA |  | tfeb Ex6 | taatacgactcactataggcgttggttttggggatcagttttagagctagaaatagc |
| tfe3a_Ex4_gRNA |  | tfe3a Ex4 | taatacgactcactata ggtgtacagcagtcagggcagttttagagctagaatagc |
| tfe3b_Ex1_gRNA |  | tfe3b Ex1 | taatacgactcactataggaggaggccgggcagccatgttttagagctagaatagc |
| tfe3b_Ex2_gRNA |  | tfe3b Ex2 | taatacgactcactataggcaactttattgccgagtggttttagagctagaatagc |
| oligo2 |  | n/a | aaaagcaccgactcggtgccactttttcaagttgataacggactagccttattttaacttgctatttctagctctaaaac |
| Tfeb_ex6_F | gDNA genotyping primers | tfeb Ex6 | tgtaaaacgacggccagtgctgattgctcgttctgtcc |
| Tfeb_ex6_R |  |  | gtgtcttggttccagatggtcaagtgc |
| Tfe3a_ex4_F |  | tfe3a Ex4 | gtgtctttaacagcagccagtctcagg |
| Tfe3a_ex4_R |  |  | tgtaaaacgacggccagtcagtcagattgactcatgaagga |
| Tfe3b_ex1_F |  | tfe3b Ex1 | tgtaaaacgacggccagtgcagcatgtcttcgagagtg |
| Tfe3b_ex1_R |  |  | gtgtcttagccagtctagacccaccttc |
| Tfe3b_ex2_F |  | tfe3b Ex2 | gtgtcttcccatctggaaaacccaac |
| Tfe3b_ex2_R |  |  | tgtaaaacgacggccagtttggagcctagattaagatgtgc |
| M13-FAM |  | n/a | 5'-FAM/tgtaaaacgacggccagt |
| tfeb_mRNA_Ex6_F | mRNA sequencing primers | tfeb Ex6 | tgtaaaacgacggccagttgcgaatctagccatcaaga |
| tfeb_mRNA_Ex6_R |  |  | gtgtctttagccatctccatcctcctg |
| tfe3a_mRNA_Ex4_F |  | tfe3a Ex4 | tgtaaaacgacggccagtgatgacatcatcagcctgga |
| tfe3a_mRNA_Ex4_R |  |  | gtgtcttgggtcagaggactttggaatc |
| tfe3b_mRNA_Ex1_F |  | tfe3b Ex1 | tgtaaaacgacggccagtgcagcatgtcttcgagagtg |
| tfe3b_mRNA_Ex1_R |  |  | gtgtcttttggagcctagattaagatgtgc |
| tfe3b_mRNA_Ex2_F |  | tfe3b Ex2 | tgtaaaacgacggccagttgcaaacccatctggaaaac |
| tfe3b_mRNA_Ex2_R |  |  | gtgtcttatctgctggacaggagttgc |
| tfe3a_WISH_FOR | WISH | tfe3a all isoforms | gcctctcgtcatctcagctc |
| tfe3a_WISH_REV |  |  | ggatccattaaccctcactaaagcctgcatctcctccagaatc |
| trypsin_WISH_FOR |  | trypsin | ggtgagcacaacattgacgtca |
| trypin_WISH_REV |  |  | ctttgccagatggtattgcaaca |
| insulin_WISH_FOR |  | ins | atctccaccaccatatccacca |
| insulin_WISH_REV |  |  | aaacggagagcattaaggcctg |
| fabp10a_WISH_FOR |  | fabp10a | gaccttcactttgtgttgagctt |
| fabp10a_WISH_REV |  |  | ctttaaagtatgaccggtacagtt |
| tfeb-fl_qPCR_FOR | qPCR primers | tfeb full length | acagtgcgcccaacagtc |
| tfeb-fl_qPCR_REV |  |  | aggctgatgatgttatcaattacg |
| tfeb-s_qPCR_FOR |  | tfeb short | aaacctatgccatcgtggag |
| tfeb-s_qPCR_REV |  |  | ctggtcatggctattgcag |
| tfeb-t_qPCR_FOR |  | tfeb total | gcatgcagaaggatgtcca |
| tfeb-t_qPCR_REV |  |  | gggcttgtctttctagctcct |
| tfe3a-fl_qPCR_F |  | tfe3a full length | tggggtccaacaaagaagag |
| tfe3a-fl_qPCR_R |  |  | cgagtcgatcagcgtcataa |
| tfe3a-s_qPCR_F |  | tfe3a short | ggttttactgccggagaatg |
| tfe3a-s_qPCR_R |  |  | ccatgccctgactgctgta |
| tfe3a-t_qPCR_F |  | tfe3a total | gaggcagaagaaagacaaccat |
| tfe3a-t_qPCR_R |  |  | ccttgttccagcgcaact |
| tfe3b-t_qPCR_F |  | tfe3b | tgtctcctgtcccgcatt |
| tfe3b-t_qPCR_R |  |  | tcgtcaatgacatcatccatct |
| ef1a_F_qPCR |  | ef1a | ttctgttacctggcaaaggg |
| ef1a_R_qPCR |  |  | ttcagtttgtccaacaccca |
| hexa_qPCR_F |  | hexa | gacccttcctttccgtatca |
| hexa_qPCR_R |  |  | cctggcatgttcaatcactc |
| ctsc_qPCR_F |  | ctsc | cgtcgtcacatgcttggat |
| ctsc_qPCR_R |  |  | catcatgttgttcgtgtacgg |
| mcln1b_qPCR_F |  | mcln1b | acacgttcgacatagaccctc |
| mcln1b_qPCR_R |  |  | taccaacttctgaaaattaagggtg |
| gba_qPCR_F |  | gba | ggagagtcaaggacagttcca |
| gba_qPCR_R |  |  | ctgactggggttcagagtgat |
| neu1_qPCR_F |  | neu1 | cgatgatggtgcaaagtctg |
| neu1_qPCR_R |  |  | gcccttactgggtggatacc |
| gla_qPCR_F |  | gla | ctggccttcgcaacaaag |
| gla_qPCR_R |  |  | acctgcgcaagtttttgtg |
| qPCR_PCNA-F |  | pcna | ggcaacatcaagctctcaca |
| qPCR_PCNA-R |  |  | tgcactggctcattcatctc |
| qPCR_ccng1-F |  | ccng1 | gtgcggagacgttttcctt |
| qPCR_ccng1-R |  |  | aagacagatgcttgggctga |
| qPCR_p21-F |  | cdk1a | ccgcatgaagtggagaaaac |
| qPCR_p21-R |  |  | acgcttcttggcttggtaga |
| qPCR_Δ113tp53-F |  | Δ113tp53 | atatcctggcgaacatttggaggg |
| qPCR_Δ113tp53-R |  |  | cctcctggtcttgtaatgtcac |
| qPCR_tp53-fl_F |  | tp53 | tggagaggaggtcggcaaaatcaa |
| qPCR_tp53-fl_R |  |  | gactgcgggaacctgagcctaaat |
| qPCR_rprmb-F |  | rprmb | tcttcgctctcgggactttca |
| qPCR_rprmb-R |  |  | cagctgcccttctccaaacaa |
| qPCR_mdm2-F |  | mdm2 | tgacaacgagaaactggtaaga |
| qPCR_mdm2-R |  |  | aaacataacctccttcatggt |
| bcl2l1-F qPCR |  | bcl2l1 | cgcatcgcagaatggatgac |
| bcl2l1-R qPCR |  |  | gccgctgcatcttttccaa |
| qPCR_baxa-F |  | baxa | ggaggcgatacgggcagtg |
| qPCR_baxa-R |  |  | ttgcgaatcaccaatgctgtg |
| qPCR_rps27l-F |  | rps27l | gactcactgagggttgctct |
| qPCR_rps27l-R |  |  | cctgccacacatcgctcata |
